# A prognostic estimation model based on mRNA-sequence data for patients with oligodendroglioma

**DOI:** 10.3389/fneur.2022.1074593

**Published:** 2022-12-14

**Authors:** Qinghui Zhu, Shaoping Shen, Chuanwei Yang, Mingxiao Li, Xiaokang Zhang, Haoyi Li, Xuzhe Zhao, Ming Li, Yong Cui, Xiaohui Ren, Song Lin

**Affiliations:** ^1^Department of Neurosurgical Oncology, Beijing Tiantan Hospital, Capital Medical University, Beijing, China; ^2^Department of Neurosurgery, Beijing Neurosurgical Institute, Capital Medical University, Beijing, China

**Keywords:** oligodendroglioma, 1p/19q codeletion, prognostic model, WHO CNS 5, mRNA-sequence

## Abstract

**Background:**

The diagnosis of oligodendroglioma based on the latest World Health Organization Classification of Tumors of the Central Nervous System (WHO CNS 5) criteria requires the codeletion of chromosome arms 1p and 19q and isocitrate dehydrogenase gene (IDH) mutation (mut). Previously identified prognostic indicators may not be completely suitable for patients with oligodendroglioma based on the new diagnostic criteria. To find potential prognostic indicators for oligodendroglioma, we analyzed the expression of mRNAs of oligodendrogliomas in Chinese Glioma Genome Atlas (CGGA).

**Methods:**

We collected 165 CGGA oligodendroglioma mRNA-sequence datasets and divided them into two cohorts. Patients in the two cohorts were further classified into long-survival and short-survival subgroups. The most predictive mRNAs were filtered out of differentially expressed mRNAs (DE mRNAs) between long-survival and short-survival patients in the training cohort by least absolute shrinkage and selection operator (LASSO), and risk scores of patients were calculated. Univariate and multivariate analyses were performed to screen factors associated with survival and establish the prognostic model. qRT-PCR was used to validate the expression differences of mRNAs.

**Results:**

A total of 88 DE mRNAs were identified between the long-survival and the short-survival groups in the training cohort. Seven RNAs were selected to calculate risk scores. Univariate analysis showed that risk level, age, and primary-or-recurrent status (PRS) type were statistically correlated with survival and were used as factors to establish a prognostic model for patients with oligodendroglioma. The model showed an optimal predictive accuracy with a C-index of 0.912 (95% CI, 0.679–0.981) and harbored a good agreement between the predictions and observations in both training and validation cohorts.

**Conclusion:**

We established a prognostic model based on mRNA-sequence data for patients with oligodendroglioma. The predictive ability of this model was validated in a validation cohort, which demonstrated optimal accuracy. The 7 mRNAs included in the model would help predict the prognosis of patients and guide personalized treatment.

## Introduction

Oligodendroglioma is a subtype of glioma with a relatively favorable prognosis compared with those of other entities (1–3). In contrast with the previous diagnostic criteria, which relied only on histopathological evidence (4), the integrated diagnosis of oligodendroglioma based on the World Health Organization Classification of Tumors of the Central Nervous System (WHO CNS 5) criteria requires the codeletion of chromosome arms 1p and 19q and isocitrate dehydrogenase gene (IDH) mutation (mut) (5). The WHO CNS 5 proposes an integrated diagnosis based on the consideration of pathological features and molecular profiles, such as IDH, 1p/19q, and TERT molecular markers. According to the new diagnostic criteria, the variant of oligoastrocytomas has been removed, along with the term “anaplastic” (5), which indicates the consensus that the histopathological classification is not adequate to predict the prognosis of tumors has been established (6). These revised diagnostic criteria for diffuse glioma are more reliable in predicting prognosis than classic histopathological methods (7).

According to prior reports, the 5 year survival rate of oligodendroglioma almost reaches 90% (8). However, we also observed that some patients showed a shorter survival time, irrespective of the treatment they received or the WHO grade. Moreover, the disparity in survival also suggests that a more objective and optimal stratification of oligodendrogliomas that is not merely dependent on histopathological evidence is urgently needed.

To identify potential prognostic factors and establish a prognostic estimation model for oligodendrogliomas in the context of the WHO CNS 5 classification, we analyzed the data from the China Glioma Genome Atlas (CGGA) database and the information of patients in the Department of Neurosurgical Oncology, Beijing Tiantan Hospital, Capital Medical University.

## Materials and methods

### CGGA data

RNA sequencing and corresponding clinical data were retrieved from the CGGA database (DataSet ID: mRNAseq_693 and mRNAseq_325) (9–13). A total of 165 glioma datasets with IDH mutation and 1p/19q co-deletion were selected and divided into two cohorts (109 in the training cohort and 56 in the validation cohort). The flowchart of the study is shown in Supplementary Figure 1. The corresponding clinical data of the patients with oligodendroglioma are summarized in Table 1.

**Table 1:** Corresponding clinical data of the patients in two cohorts.

**Variables**	**Training cohort** **(*n* = 109)**	**Validation cohort** **(*n* = 56)**	***P*-value**
Survival status			0.9161
Alive	77	40	
Dead	32	16	
PRS status			0.5614
Primary	65	36	
Recurrent	44	20	
Gender			0.0709
Male	50	34	
Female	59	22	
Age			0.2618
≤ 60	103	55	
>60	6	1	
Adjuvant therapy			0.3429
Radiotherapy + Chemotherapy	52	26	
Chemotherapy only	17	25	
Radiotherapy only	30	3	
None	10	2	

### Quantitative reverse transcription polymerase chain reaction using clinical samples

Tumor tissues of the patient collected from the Department of Neurosurgical Oncology of Beijing Tiantan Hospital were used for qRT–PCR. We chose patients with IDH mut and 1p/19q-codeleted oligodendroglioma and obtained their cryopreserved tumor samples along with the corresponding clinical data. Total RNA was isolated from tumor tissues. M-MLV reverse transcriptase (TaKaRa Bio, Japan) was used to synthesize cDNA. qRT–PCR was performed using a Roche LightCycler^®^ 480II system (Roche, Switzerland). GAPDH was used as an internal reference to quantify the mRNAs. qRT–PCR assays were performed in triplicate. The primer and probe sequences of the genes used for qRT–PCR analysis are listed in Supplementary Table 1. Using patients with a survival time of more than 5 years as the control group, cycle threshold (CT) values were converted to relative expression to validate the expression differences.

### Screening of differentially expressed mRNAs and calculating risk score

Since many low-grade glioma studies have used 5-year survival as an indicator of long-term survival (14, 15), we adopted 1,825 days (≈5 years) as the cutoff point to classify the length of patient survival time. The DESeq2 package was used to filter the differentially expressed mRNAs (DE mRNAs) between the long survival (>1,825 days) and short survival patients (≤ 1,825 days) in the training cohort with the criteria of an |log_2_-fold-change| ≥ 1.0 and a false discovery rate of the *p* < 0.001. The package made use of empirical Bayes techniques to estimate priors for log-fold-change and dispersion and to calculate posterior estimates for these quantities (16). The least absolute shrinkage and selection operator (LASSO) regression analysis was used to select the most useful predictive mRNAs from the mRNA-sequence and survival data (17). The risk score was calculated for each patient and was used to divide patients into two different groups (high-risk and low-risk). Survival analysis conducted with the DE mRNA accompanied by the corresponding clinical data was performed using the survival package. The ROCR, glmnet, and caret packages were used to assess the accuracy of the prediction of survival by risk level. Receiver operating characteristic (ROC) curves were generated to validate the accuracy of the risk score for predicting patients' OS.

### Establishment of a prognostic model

Clinical data were combined with the risk scores of patients. A multivariate Cox proportional hazards model was constructed using the significant parameters identified by univariate analysis of all factors in the combined data. The multivariable Cox regression analysis included the following factors: age (>60 or not), primary-or-recurrent status (PRS) type, and risk level. A prognostic model was generated to predict the survival time of patients suffering from oligodendroglioma. To provide the clinician with an intuitive method for assessing the possibility of survival at a specific time, we established a prognostic nomogram model prepared by binary logistics regression analysis in the training cohort.

### Apparent performance of the prognostic model in the training cohort

The Hosmer–Lemeshow test was used to verify the accuracy of the prognostic model. A calibration curve was plotted to visualize the results, and the ability of the model to predict a prognosis of death was evaluated by ROC. The model was subjected to bootstrapping validation (1,000 bootstrap resamples) to plot the calibration curves. Harrell's concordance index (C-index) was used to calculate the degree of discrimination between the predicted value and the observed value.

### Independent validation of the prognostic model

The performance of the prognostic model was tested in a validation cohort. The Cox regression formula established in the training cohort was applied to all patients in the validation cohort. The C-index and calibration curve were used to evaluate the accuracy of our prediction model.

### Statistical analysis

Statistical analysis was conducted using R software (version 4.1.3. http://www.r-project.org). The packages in R that were used in this study are reported in Supplementary Table 2. The reported statistical significance levels were all obtained from two-sided tests, and a *p* < 0.05 was considered statistically significant.

## Results

### Identification of DE mRNAs

A total of 165 oligodendroglioma datasets from the CGGA mRNA-sequencing database were selected, including 109 datasets in the training cohort and 56 datasets in the validation cohort. The baseline of the two cohorts showed no statistically significant differences (Table 1). After normalization, 88 mRNAs were identified to be significantly differentially expressed (|log_2_-fold-change| ≥ 1.0 and false discovery rate of *p* < 0.001) between the long-survival and short-survival patients in the training cohort. The expression levels of the DE mRNAs are presented in Supplementary Figure 2.

### LASSO gene selection and risk score calculation

**Figure 1:**
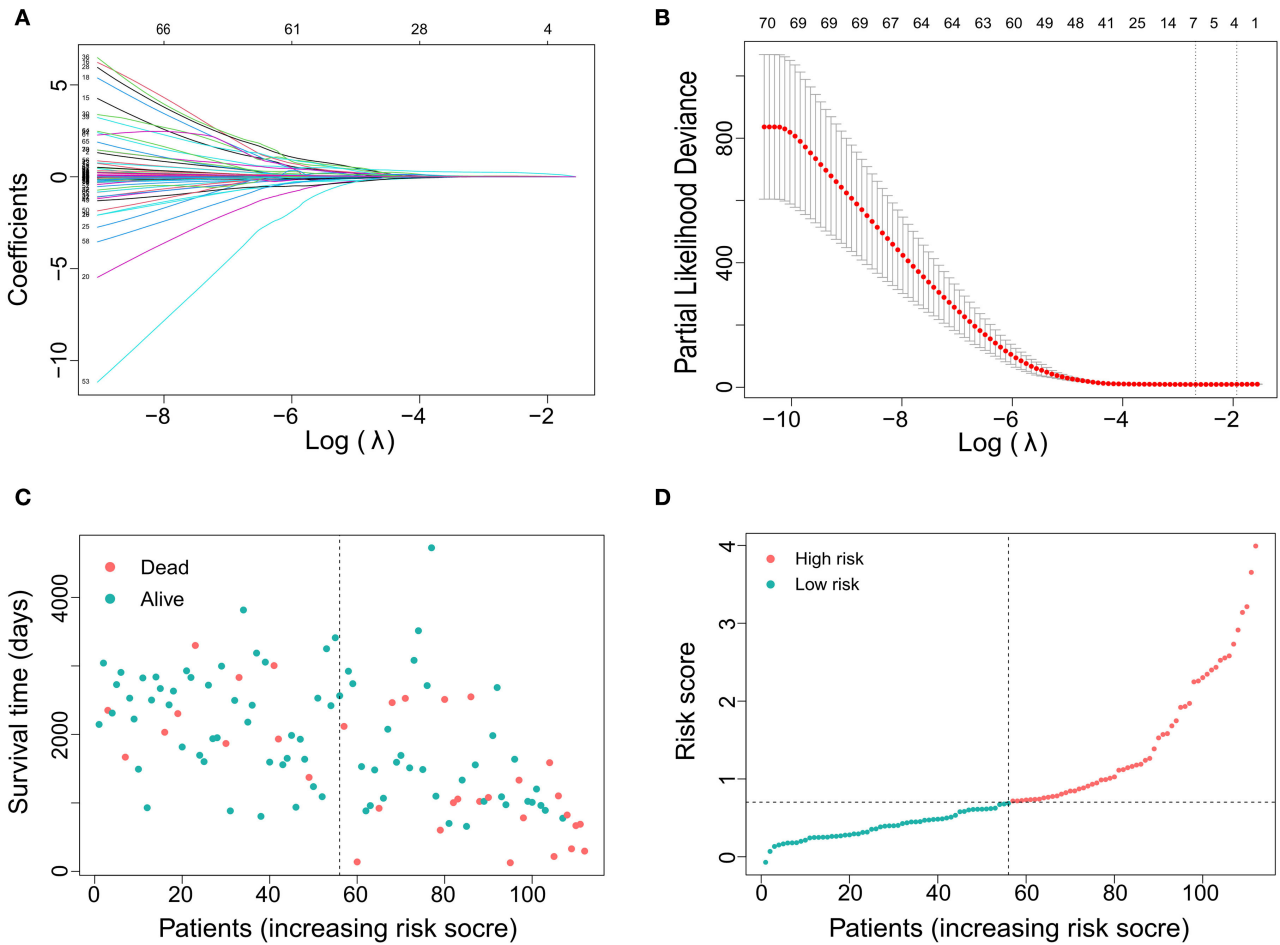
Gene selection using the least absolute shrinkage and selection operator (LASSO) binary logistic regression model. **(A)** LASSO coefficient profiles of the 88 DE RNAs. A coefficient profile plot was produced against the log (lambda). **(B)** The area under the receiver operating characteristic (AUC) curve was plotted vs. log(l). Dotted vertical lines were drawn at the optimal values using the minimum criteria and the 1 standard error of the minimum criteria (the 1-SE criteria). **(C)** Distribution of survival time showing that survival time decreases with increasing risk score and the incidence of time to death increases. **(D)** Distribution curve showing the distribution of risk score.

In total, 88 DE mRNAs were reduced to 7 mRNAs (LOXL2, APLN, TROAP, NUF2, CTHRC1, SLC12A5, and ANGPT2) in the LASSO regression model using the minimum criteria for risk factors (Figures 1A,B). These mRNAs were features with non-zero coefficients in the LASSO logistic regression model. This approach can be used not only to select mRNAs based on the strength of their univariate association with survival but also to calculate a risk score and perform risk stratification. The distribution of risk scores is shown in Figures 1C,D. The distribution showed that as the risk score increased, the survival time of patients decreased, and the mortality rate increased. The area under the ROC curve (AUC) was 0.679, indicating that the risk score had acceptable prediction accuracy for survival.

### Establishment of the individualized prognostic model

**Figure 2:**
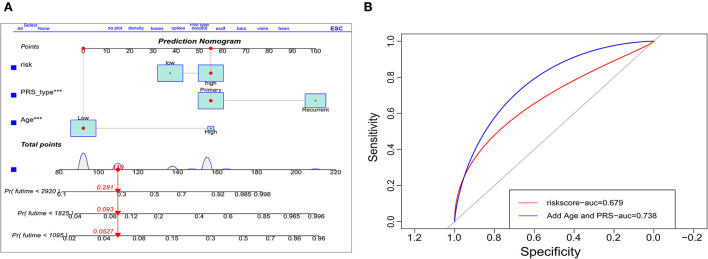
Validation of the prognostic model. **(A)** Dynamic nomogram plots were generated to visualize the prognostic model, and **(B)** ROC curves demonstrate the predictive ability of risk scores (AUC = 0.679) vs. the use of risk level and age and primary-or-recurrent status (PRS)-type (AUC = 0.738).

**Figure 3:**
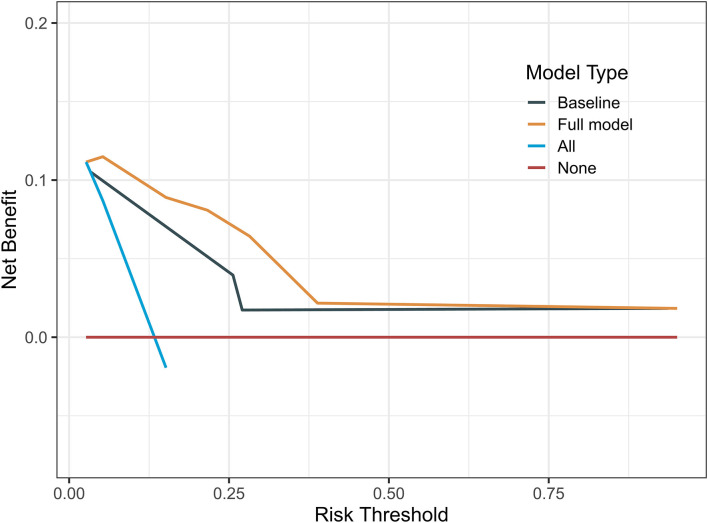
Decision curves of the prognostic models showed that the prognosis model based on risk scores and baseline data was more accurate.

**Figure 4:**
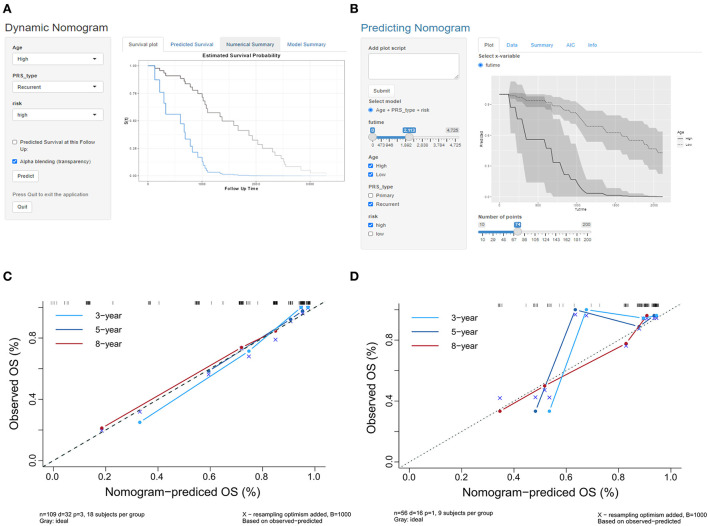
**(A,B)** Scripts were generated to predict the probability of survival for a patient at a specific time point. **(C)** The calibration curves in the training cohort show that the predicted values at 3, 5, and 8 years are close to the observed values. **(D)** The calibration curves at 3, 5, and 8 years showed that the prediction accuracy of the prognosis model was acceptable, and the model had the highest accuracy in predicting 8 year survival.

The indicators with *p* < 0.05 in multivariate analysis (age, PRS status, and risk level) were selected as components of the established prognostic model. Age > 60 years, recurrent tumors, and high-risk level predicted a worse prognosis in patients with oligodendroglioma. With these parameters, a prognostic model was established. A nomogram was generated to present the results (Figure 2A). The ROC curve indicated that the predictive ability of the model was improved after age and PRS status was included (AUC: 0.738 vs. 0.679) (Figure 2B). Compared with the prognostic model merely based on baseline data, the predictive value of the combined model showed higher accuracy (Figure 3). At the same time, based on the prognostic model, the DynNom package and the shinyPredict package were used to generate scripts to individually predict the prognosis of patients with oligodendroglioma (Figures 4A,B).

### Evaluation of the prognostic model in the training cohort

The calibration curves of the prognostic model for the probability of survival at 3, 5, and 8 years showed similarities in the observed and predicted values in the training cohort (Figure 4C). The Hosmer–Lemeshow test suggested that there was no departure from perfect fit (*p* = 0.9997; Table 2). The prognostic model yielded a C-index of 0.912 (95% CI, 0.679–0.981) in the training cohort, which implied that the model had good accuracy for predicting prognosis.

**Table 2:** The predicted value in the Hosmer–Lemeshow test is close to the observed value.

**Interval**	**Alive**	**Dead**	**Predicted alive**	**Predicted dead**
[0.118, 0.125)	38	6	38.829471	5.170529
[0.125, 0.153)	15	2	14.392965	2.607035
[0.153, 0.422)	6	3	5.205934	3.794066
[0.422, 0.498)	16	17	16.571630	16.428370
[0.498, 0.889]	2	4	2.000000	4.000000

### Independent validation of the prognostic model

The calibration curves of the prognosis model showed good agreement between the predictions and observations in the validation cohort (Figure 4D). The Hosmer–Lemeshow test yielded a non-significant *p*-value (*p* = 0.8921), and the C-index of the nomogram for the prediction of survival was 0.778 (95% CI, 0.769–0.787). The prognosis model predicted a good consistency between the probability of death among patients and the actual percentage of death in the observed population.

### Validation of RNA expression differences between tumor tissues of patients

**Figure 5:**
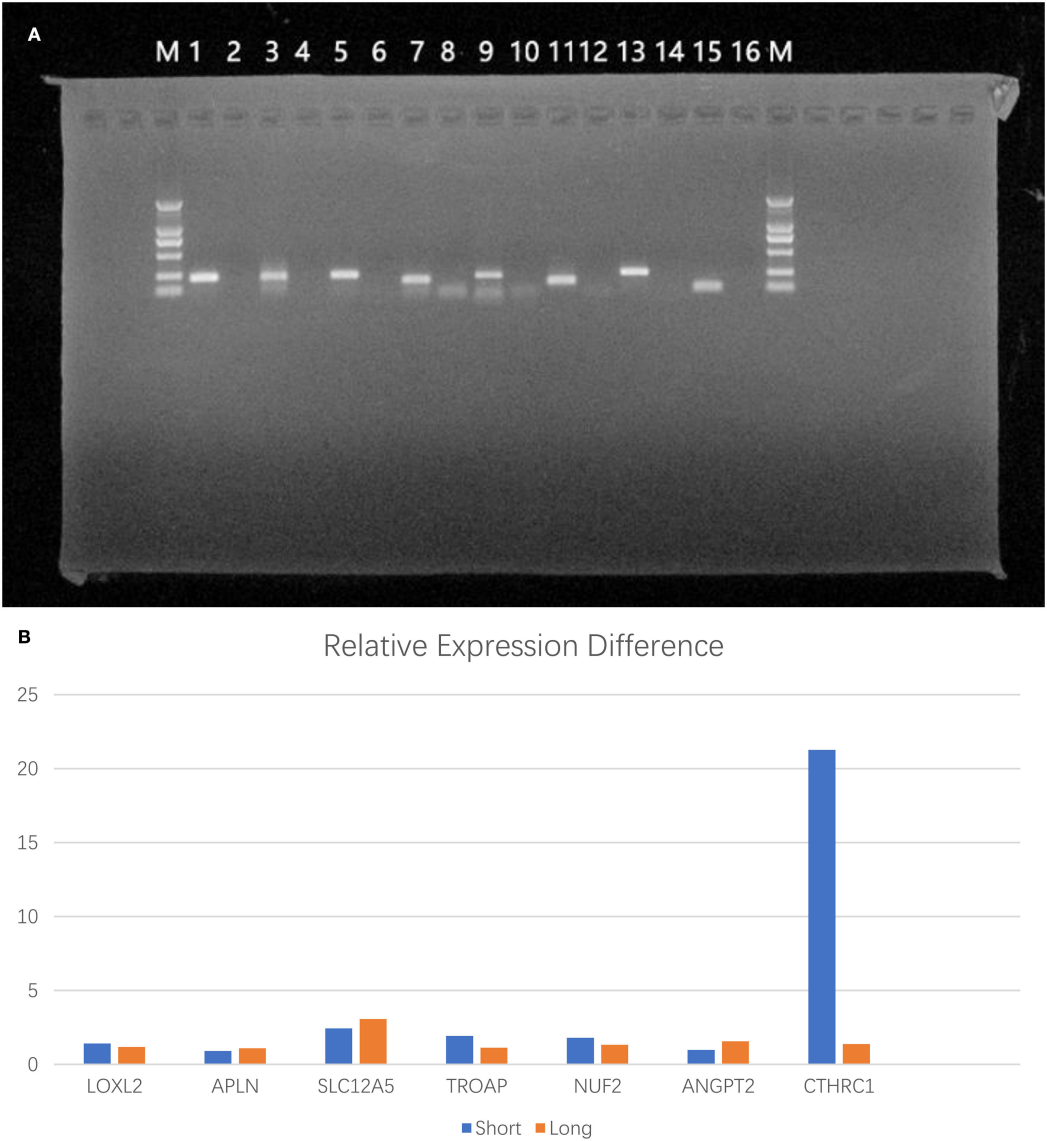
Validation of RNA expression differences between tumor tissues of patients. **(A)** Electropherogram of PCR products (M: DL2000; 1: GAPDH; 2: GAPDH negative control; 3: LOXL2; 4: LOXL2 negative control; 5: APLN; 6: APLN negative control; 7: SLC12A5; 8: SLC12A5 negative control; 9: TROAP; 10: TROAP negative control; 11: ANGPT2; 12: negative control; 13: NUF2; 14: NUF2 negative control; 15: CTHRC1; 16: CTHRC1 negative control). **(B)** The differences in mean relative gene expression between the two groups.

Samples from the long-survival and short-survival patients were included for qRT–PCR to calculate the relative expression levels. We sought to validate the differential expression of the seven target genes (LOXL2, APLN, SLC12A5, TROAP, NUF2, ANGPT2, and CTHRC1) among these samples (Figure 5A). Unfortunately, although the differences in the mean relative gene expression between groups were consistent with expectations, except for APLN and ANGPT2, there were no statistically significant differences in gene expression between the two groups (Figure 5B). Due to the limitations of preservative methods and the long survival time of patients with oligodendroglioma, a suitable number of samples was difficult to obtain, which resulted in an insufficient sample size.

## Discussion

Using the clinical and RNA-seq information contained in the CGGA database, we identified 7 mRNAs that could be used to predict prognosis. Then, we established a prognosis model for patients with oligodendroglioma based on seven selected mRNAs, which showed high predictive accuracy. Accurate prognosis prediction is an important component of the individualized treatment of tumors (18). Several studies aimed to find more accurate strategies to predict the prognosis of patients with oligodendroglioma (19, 20). Cao et al. analyzed the information of 4,568 patients with oligodendroglioma and established a prognostic model based on the Surveillance, Epidemiology, and End Results (SEER) database. Final results showed that radiotherapy, age, tumor location, grade, and surgical resection were independent prognostic factors of oligodendroglioma. However, they only focused on oligodendrogliomas diagnosed by histopathological criteria, and the molecular pathology factors have been neglected (19).

In addition to assessments of pathology physiological changes, consideration of the patients in daily life was also a necessary part of estimating the survival of patients with cancer (21). The survival analysis of high-grade oligodendroglioma by Liu et al. was more comprehensive and more detailed in the collection of clinical information (20). Family circumstances were also included in the survival analysis. This prognosis model was consistent with the International Classification of Functioning, Disability, and Health guidelines. Their model introduced new metrics for the establishment of prognostic models. The disadvantage of this model was that it lacked molecular pathological information for a more accurate diagnosis of patients.

Unlike previous studies, our study is one of the rare prognostic studies of oligodendroglioma combined with clinical and molecular data. In our study, the AUC value of the ROC curve of the prognostic model that integrated clinical and molecular data was higher than that of the prognostic model that contained only molecular data, and the C-index value of our prognosis model was higher than that of the Cao L study, which contained only clinical information. These results demonstrated that the integration of clinical and molecular factors could predict prognosis more accurately. In the future, more clinical information can be collected from patients for larger and more detailed prognostic analysis to establish a more accurate individualized prognostic prediction model.

Biomarkers, as prognostic indicators, are always of great interest to researchers. Some studies regarding the molecular markers associated with the survival of patients with oligodendroglioma have aimed to stratify oligodendroglioma, with an attempt to identify possible therapeutic targets and improve the survival of patients (22–26). Several previous studies that aimed to identify prognostic indicators of oligodendroglioma proposed the use of CDKN2A/B, PTEN, NOTCH1, and other biomarkers as classification criteria to reclassify oligodendroglioma, but there is no consistent conclusion (22–25). These prognostic indicators that were assessed in previous studies were discussed based on histology-confirmed oligodendrogliomas rather than an integrated diagnosed oligodendroglioma. For oligodendroglioma with IDH mutation and 1p/19q codeletion, our previous work showed that patients with oligodendroglioma with 1q/19p copolysomy had a worse prognosis (26).

In this study, we found that 7 DE mRNAs, namely, LOXL2, APLN, TROAP, NUF2, CTHRC1, SLC12A5, and ANGPT2, showed the greatest impact on prognosis. These mRNAs may play a vital role in the tumorigenesis of oligodendroglioma. Previous studies have shown that LOXL2, a member of the lysyl oxidase (LOX) family, not only promotes glioma cell proliferation, migration, and invasion and induces the epithelial-to-mesenchymal transition (EMT) process but also reduces the sensitivity of glioma cells to temozolomide (TMZ) (27). APLN is activated by VEGF signaling and hypoxia-responsive elements in the APLN promoter, stimulates angiogenic sprouting, and plays a necessary and sufficient role in tumor angiogenesis (28). TROAP activates the Wnt/β-catenin pathway and upregulates the expression of its downstream targets to play a tumor-promoting role (29). ANGPT2 activates angiogenesis through VEGFA, normalizes tumor blood vessels, and promotes the malignant transformation of glioblastoma (30, 31). NUF2 has a potential role in glioma growth and TMZ resistance (32). The CTHRC1 gene contributes to tissue repair in vascular remodeling in response to injury by limiting collagen matrix deposition and promoting cell migration (33). In addition to the mRNAs with increased expression mentioned above, which are related to cell differentiation and proliferation, the decreased expression of SLC12A5 aroused our interest. SLC12A5 (encoding the KCC2 protein) acts to stabilize nerve cell potential, and its reduced expression correlates with the development of epilepsy (34–39). Consistent with our findings, Yang and Gao et al. found that the expression of SLC12A5 in patients with shorter survival time was significantly lower than that in patients with longer survival (40). On the one hand, this finding may be explained by the fact that epilepsy is associated with IDH1 mutations in low-grade gliomas (41), and IDH mutations predict better glioma prognosis. On the other hand, seizures may cause some patients to seek treatment more actively, which may result in a better prognosis (42, 43). These DE mRNAs can be used not only to predict the prognosis of patients with oligodendroglioma but also to provide directions for potential therapeutic targets of oligodendroglioma. ISL2, a nuclear and chromatin-associated transcription factor (44), regulates the transcription of ANGPT2 by binding to the ANGPT2 promoter. When ISL2 expression was found to be reduced, oligodendroglioma cell proliferation was reduced. TMZ combined with anti-ISL2 therapy may be effective in oligodendroglioma *in vitro* and tumor-bearing animal models (30). For this possible treatment, more clinical studies are required to study its safety and efficiency.

In our study, we established a prognosis model for patients with oligodendroglioma. The model might be used to assign individualized treatment plans for patients with oligodendroglioma. Although excellent predictive models were identified, some limitations still existed in our study. Constrained by limited conditions, the study only contained data from CGGA and lacked verification performed using other databases. Most of the cases in the CGGA database are patients treated in our hospital, so there may have been some bias in the selection of patients included in the analysis. At the same time, our study was a retrospective study, and the treatments that patients received were not strictly consistent, which may decrease the power of our model. Additionally, studies of molecular mechanisms are needed in the future to explain the differential expression of mRNAs among patients with oligodendroglioma.

## Conclusion

We established an individualized prognostic model for patients with 1p/19q codeleted and IDH mutant oligodendroglioma based on mRNA seq data. The model would help predict the prognosis of patients and guide personalized treatment.

## Data availability statement

The original contributions presented in the study are included in the article/Supplementary material, further inquiries can be directed to the corresponding author.

## Ethics statement

The studies involving human participants were reviewed and approved by Medical Ethics Committee of Tiantan Hospital, Beijing Tiantan Hospital. The patients/participants provided their written informed consent to participate in this study.

## Author contributions

QZ and SL: conception and design. QZ, MingxL, XZhan, XZhao, YC, and MingL: collection and assembly of data. QZ, HL, SS, XR, and CY: data analysis and interpretation. QZ: cell biological experiments and manuscript writing. All authors: final approval of manuscript.

## Supplementary material

Supplementary Figure 1 Flowchart of the study.

Supplementary Figure 2 Heatmap of differentially expressed mRNAs (DE mRNAs).

Supplementary Table 1 Information of primer sequences of the genes used for qRT–PCR analysis.

Supplementary Table 2 R packages used in the study.
