# Supplementary figures and images for: A prognostic estimation model based on mRNA-sequence data for patients with oligodendroglioma
Source: Front Neurol. 2022 Dec 14;13:1074593. doi: 10.3389/fneur.2022.1074593 (PMC9795846; doi:10.3389/fneur.2022.1074593)

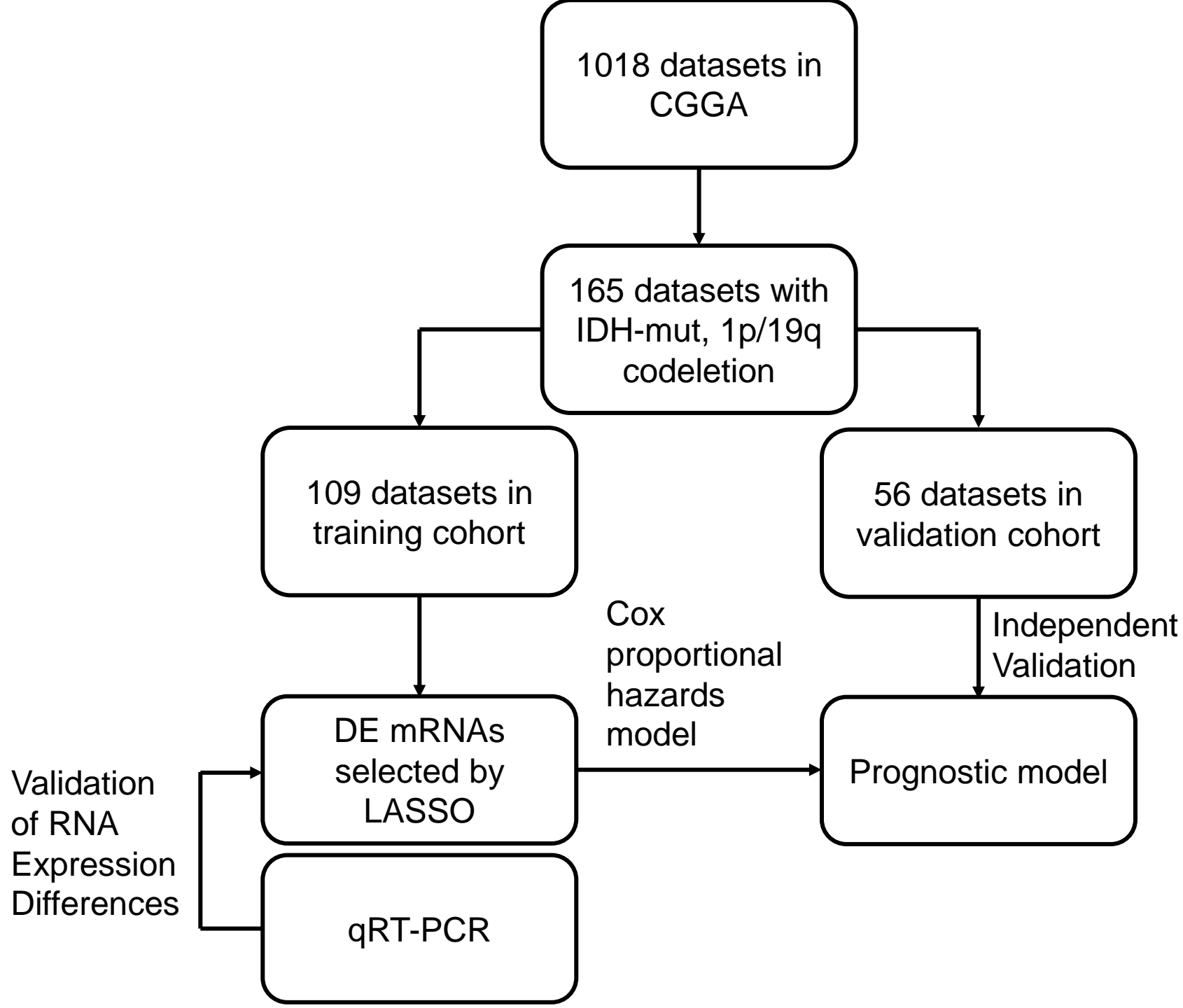

Supplement: Supplementary Figure 1 — Flowchart of the study. [file Image_1.PDF]

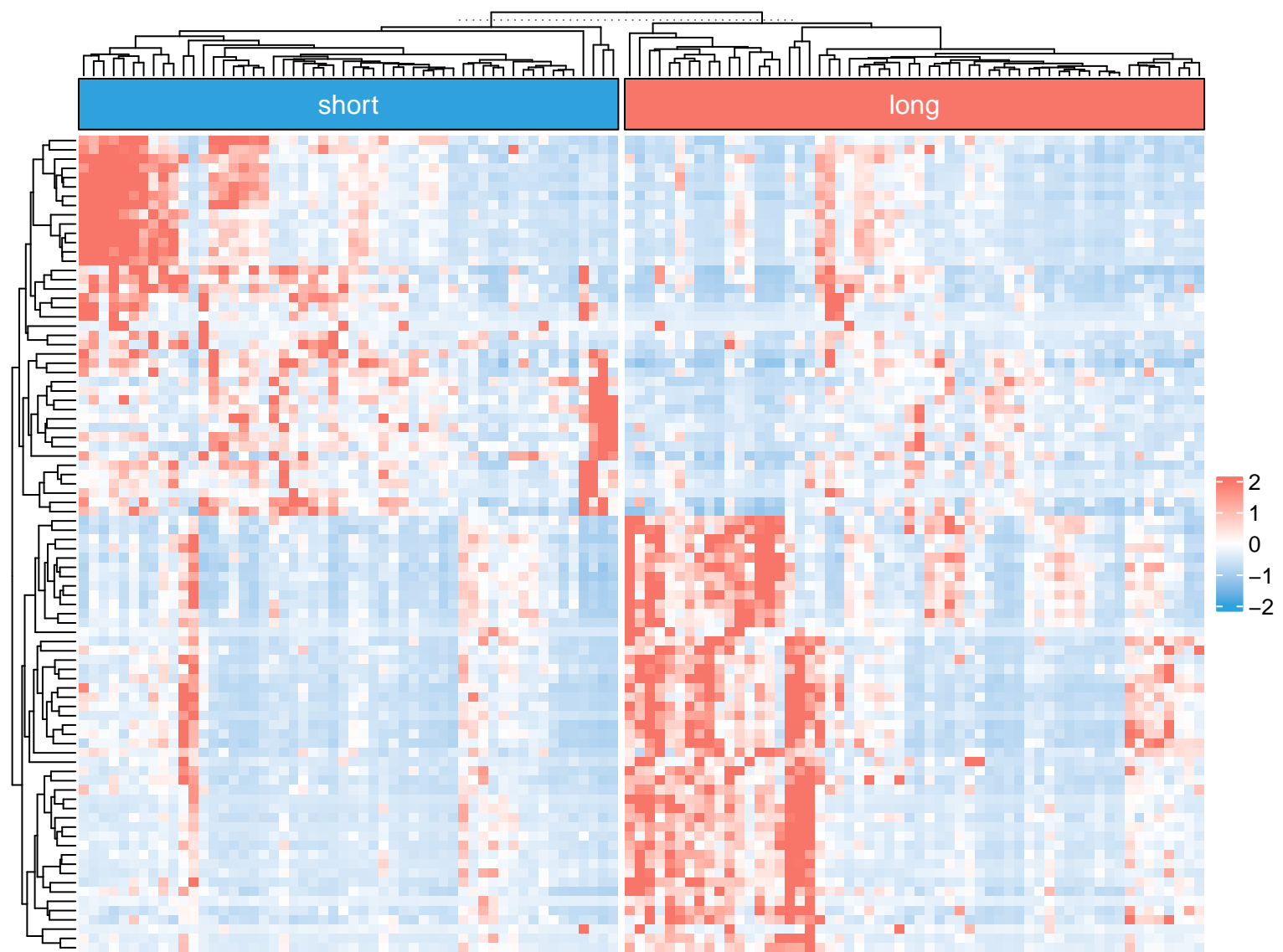

Supplement: Supplementary Figure 2 — Heatmap of differentially expressed mRNAs (DE mRNAs). [file Image_2.PDF]
